# Creatinine- versus cystatin C-based renal function assessment in the Northern Manhattan Study

**DOI:** 10.1371/journal.pone.0206839

**Published:** 2018-11-14

**Authors:** S. Ali Husain, Joshua Z. Willey, Yeseon Park Moon, Mitchell S. V. Elkind, Ralph L. Sacco, Myles Wolf, Ken Cheung, Clinton B. Wright, Sumit Mohan

**Affiliations:** 1 Department of Medicine, Division of Nephrology, College of Physicians and Surgeons, Columbia University Medical Center, New York, New York, United States of America; 2 Department of Neurology, College of Physicians and Surgeons, Columbia University, New York, New York, United States of America; 3 Department of Epidemiology, Mailman School of Public Health, Columbia University, New York, New York, United States of America; 4 Departments of Neurology and Public Health Sciences, Leonard M. Miller School of Medicine, the McKnight Brain Institute and the Neuroscience Program, University of Miami, Miami, Florida, United States of America; 5 Department of Medicine, Division of Nephrology, Duke University School of Medicine, Durham, North Carolina, United States of America; 6 Department of Biostatistics, Mailman School of Public Health, Columbia University, New York, New York, United States of America; University of Colorado Denver School of Medicine, UNITED STATES

## Abstract

**Background:**

Accurate glomerular filtration rate estimation informs drug dosing and risk stratification. Body composition heterogeneity influences creatinine production and the precision of creatinine-based estimated glomerular filtration rate (eGFR_cr_) in the elderly. We compared chronic kidney disease (CKD) categorization using eGFR_cr_ and cystatin C-based estimated GFR (eGFR_cys_) in an elderly, racially/ethnically diverse cohort to determine their concordance.

**Methods:**

The Northern Manhattan Study (NOMAS) is a predominantly elderly, multi-ethnic cohort with a primary aim to study cardiovascular disease epidemiology. We included participants with concurrently measured creatinine and cystatin C. eGFR_cr_ was calculated using the CKD-EPI 2009 equation. eGFR_cys_ was calculated using the CKD-EPI 2012 equation. Logistic regression was used to estimate odds ratios and 95% confidence intervals of factors associated with reclassification from eGFR_cr_≥60ml/min/1.73m^2^ to eGFR_cys_<60ml/min/1.73m^2^.

**Results:**

Participants (n = 2988, mean age 69±10yrs) were predominantly Hispanic, female, and overweight/obese. eGFR_cys_ was lower than eGFR_cr_ by mean 23mL/min/1.73m^2^. 51% of participants’ CKD status was discordant, and only 28% maintained the same CKD stage by both measures. Most participants (78%) had eGFR_cr_≥60mL/min/1.73m^2^; among these, 64% had eGFR_cys_<60mL/min/1.73m^2^. Among participants with eGFR_cr_≥60mL/min/1.73m^2^, eGFR_cys_-based reclassification was more likely in those with age >65 years, obesity, current smoking, white race, and female sex.

**Conclusions:**

In a large, multiethnic, elderly cohort, we found a highly discrepant prevalence of CKD with eGFR_cys_ versus eGFR_cr_. Determining the optimal method to estimate GFR in elderly populations needs urgent further study to improve risk stratification and drug dosing.

## Introduction

Accurate and reliable glomerular filtration rate (GFR) estimation has enabled the identification and classification of renal dysfunction in a manner that could not be done with the use of serum creatinine values in isolation.[1–3] The calculation of estimated GFR (eGFR) using creatinine, an endogenous amino acid derivative of muscle cells, can inform drug dosing and guide risk stratification.[4–6] By taking into account factors that impact creatinine generation, eGFR equations are able to provide an assessment of GFR without the cost or complexity associated with GFR “measurement” using exogenous substances such as inulin or iohexol.[7]

However, the use of creatinine is imperfect: because creatinine generation is dependent on muscle mass, factors that influence body composition, including age, sex, and race, adversely impact the reliability of creatinine-based GFR estimation.[8] Due to these limitations, Kidney Disease Improving Global Outcomes (KDIGO) guidelines recommend confirming creatinine-based chronic kidney disease (CKD) diagnosis using an alternative method of GFR estimation in select groups.[9] In the United States, the widely-used MDRD (Modification of Diet in Renal Disease) and CKD-EPI (Chronic Kidney Disease Epidemiology Collaboration) equations were initially developed from cohorts with a low prevalence of socio-demographic characteristics that may affect serum creatinine (mean age 50.6 years, 88% White and mean age 47 years, 95% White or Black race respectively).[10, 11]

Age-related body composition changes in the elderly lead to a decline in the relative proportion of muscle mass and a corresponding decrease in creatinine production. In this setting, stable renal function is reflected by decreasing serum creatinine concentrations over time, and thus a decline in GFR may not be reflected by significant increases in serum creatinine.[12] The uncertainty around creatinine-based estimates’ ability to adequately estimate GFR across a variety of body compositions, particularly at the extremes of age, has led to interest in using alternative biomarkers. Cystatin C is an endogenous protease inhibitor produced at a stable rate by most nucleated cells, and its generation has less inter-person variability than that of creatinine, especially as related to ethnicity, age, or sex.[8, 13, 14] Serum cystatin C values have been shown to be predictive of mortality, and cystatin C-based GFR estimating (eGFR_cys_) equations have been shown to outperform those using creatinine-based eGFR (eGFR_cr_) in prediction of CKD-associated morbidity and mortality.[10, 15–20] Notably, although eGFR_cys_ does not significantly outperform eGFR_cr_ in accuracy of GFR quantification in the general population, there appears to be a greater advantage in the elderly.[21, 22] Further, while efforts to develop new models for GFR estimation in the elderly have focused on European populations, the relative performance of creatinine- and cystatin-based equations remains unclear in Hispanics, which is concerning given that this is the fastest growing segments of the United States population.[21, 23, 24]

The objectives of this study are to determine the concordance of cystatin- and creatinine-based CKD diagnosis in the Northern Manhattan Study (NOMAS) cohort, an elderly, racially/ethnically diverse cohort in northern Manhattan. Given that these demographic characteristics were not well represented during the development of commonly used eGFR equations, we hypothesized that there would be significant discordance in CKD prevalence when using the two different GFR estimation methods.

## Methods

### Cohort

NOMAS is a prospective study with a primary aim to evaluate cardiovascular disease risk factors in an urban, racially/ethnically diverse community in northern Manhattan. Participants were eligible for enrollment if they were age≥40 years, had no prior history of stroke, had a telephone, and resided in Northern Manhattan for 3 months prior to completion of an enrollment phone interview.[25] All participants had serum creatinine measured at enrollment, and a subsample (n = 2988, 90.6%) had cystatin C measurements on stored blood samples as part of an ancillary study. All procedures performed were approved by and conducted in accordance with the ethical standards of the Columbia University Medical Center’s Institutional Review Board. Written informed consent was obtained from all participants.

### Assessment of renal function, demographic variables, and risk factors

Blood samples were obtained during baseline enrollment (1993–2001). Creatinine and cystatin C values were measured on samples obtained at the same time point (baseline enrollment) for each patient. Laboratory testing was performed at Columbia University and the University of Miami. Serum creatinine (mg/dL) measurements used Olympus instrumentation with a Jaffe-based method. Although initial creatinine concentrations were measured prior to IDMS standardization, creatinine was re-measured in 100 samples stored at -80°C using an IDMS-traceable method for creatinine measurement in order to develop a correction factor similar to what has been done successfully by other cohorts.[26, 27] The mean difference between standardized and non-standardized creatinine was -0.056±0.079mg/dL (r = 0.98). (S1 and S2 Figs). In the absence of a meaningful difference, a calibration factor was not applied prior to using creatinine values for GFR estimation using the CKD-EPI 2009 equation.[28] However, a sensitivity analysis was performed by repeating the primary analysis using creatinine values after calibration factor application. Cystatin C (mg/L) was measured on samples (84% plasma, 14% serum, 2% unspecified) stored at -80°C using Roche Diagnostics Cystatin Reagents on a Roche analyzer, standardized against ERM-DA471/IFCC reference material (intra-assay coefficient of variation (CV) 2.8% and interassay CV 4.1%; reference range 0.5–1.3 mg/L). The eGFR_cys_ estimation was based on the CKD-EPI 2012 equation.[10]

Height and weight were measured during the initial patient assessment; overweight was defined as BMI 25-30kg/m^2^ and obesity as BMI>30 kg/m^2^. Race, ethnicity, and smoking status were self-reported. Hypertension was defined as systolic blood pressure >140mmHg or diastolic blood pressure >90mmHg based on the mean of two blood pressure measurements or the patient's self-report of a history of hypertension/antihypertensive use. Diabetes mellitus was defined by self-report, fasting blood glucose level >126mg/dL, or insulin/oral hypoglycemic use. Hypercholesterolemia was based on self-report, lipid lowering therapy use, or fasting total cholesterol level >240mg/dL.

### Statistical analysis

Calculated eGFR_cr_ and eGFR_cys_ were dichotomized at a clinical cutoff of 60ml/min/1.73m^2^ consistent with standard GFR-based definitions of CKD (i.e., eGFR<60ml/min/1.73m^2^ = CKD).[9] Discordance was defined as CKD diagnosis by only one of the two estimates. A Bland-Altman analysis was used to assess agreement between eGFR_cr_ and eGFR_cys_ by plotting the difference between the two estimates (eGFR_cr_—eGFR_cys_) against their mean ([eGFR_cr_ + eGFR_cys_]/2) for each participant. Reclassification was defined as change in eGFR_cr_-based CKD diagnosis when using eGFR_cys_ (ie. eGFR_cr_≥60ml/min/1.73m^2^ with eGFR_cys_<60ml/min/1.73m^2^ OR eGFR_cr_<60ml/min/1.73m^2^ with eGFR_cys_≥60ml/min/1.73m^2^). Given the uncertainty regarding which GFR estimate is more accurate in populations like ours and the absence of measured GFR data, we chose this definition of reclassification because creatinine-based GFR estimation is currently widely used in standard practice. We did, however, note that very few participants had eGFR_cr_<60ml/min/1.73m^2^ with eGFR_cys_>60ml/min/1.73m^2^ and therefore focused our analysis of reclassification on participants with eGFR_cr_≥60ml/min/1.73m^2^.

We first assessed reclassification among those with eGFR_cr_≥60ml/min/1.73m^2^. The proportion of reclassification was calculated and compared by baseline demographics and comorbidities using multivariate logistic regression with indication of reclassification [1 for reclassification (eGFR_cys_<60ml/min/1.73m^2^) and 0 for no reclassification (eGFR_cys_≥60ml/min/1.73m^2^)] as a dependent variable. We calculated the odds ratio and 95% confidence interval (OR, 95% CI) for the association with the proportion of reclassification. A similar analysis was not performed on those with eGFR_cr_<60ml/min/1.73m^2^ because only a small number (n = 43) were reclassified with eGFR_cys_≥60ml/min/1.73m^2^; these participants were manually reviewed.

We performed two sensitivity analyses. First, we rechecked the proportion of reclassification using eGFR_cr_ recalculated after applying a calibration factor to SCr values (based on rechecking 100 samples using an IDMS-traceable method for creatinine, as above). Next, we rechecked the proportion of reclassification only among participants with age<65 years who self-identified as white race, a population more similar to the group in which the CKD-EPI equations were originally developed. Finally, we calculated eGFR using the combined creatinine-cystatin CKD-EPI 2012 equation (eGFR_cr-cys_) to determine the difference in eGFR-based CKD prevalence using each of the three estimates. Analyses were conducted using SAS version 9.3 (Cary, NC) and R version 3.5.0.

## Results

Among the 3298 NOMAS cohort participants, 2988 (91%) had both serum creatinine and cystatin C measured at the same time point and were included in our analysis. The mean age of the final cohort was 69±10 years, with 61% individuals older than 65 years at the time of data collection. Participants were predominantly Hispanic (53%), female (63%), and either overweight (41%) or obese (28%) (Table 1).

**Table 1:** Baseline characteristics of cohort.

Age (years), mean (sd)	69 (10.2)
**Sex**	
Female	1887 (63%)
Male	1101 (37%)
**Race**	
White	619 (21%)
Black	725 (24%)
Hispanic	1577 (53%)
Other	67 (2%)
**BMI (kg/m**^**2**^**)**	
<20	152 (5%)
20–24	782 (26%)
25–30	1232 (41%)
>30	822 (28%)
**Smoking Status**	
Never Smoker	1404 (47%)
Former Smoker	1084 (36%)
Current Smoker	498 (17%)
**Comorbidities**	
Diabetes Mellitus	634 (21%)
Hypertension	2196 (73%)
Any Cardiac Disease	704 (24%)
**Estimated GFR (ml/min/1.73m**^**2**^**)**	
eGFR_cr_, mean (sd)	75 ± 19
eGFR_cys_, mean (sd)	52 ± 17
**Antihypertensive Use**	
Diuretic	465 (16%)
Beta blocker	355 (12%)
Calcium channel blocker	596 (20%)
ACE inhibitor	491 (16%)

sd = standard deviation, BMI = body mass index, GFR = glomerular filtration rate, eGFR_cr_ = creatinine-based estimated GFR using the CKD-EPI 2009 equation, eGFR_cys_ = cystatin C-based estimated GFR using the CKD-EPI 2012 equation, ACE = angiotensin converting enzyme

**Fig 1:**
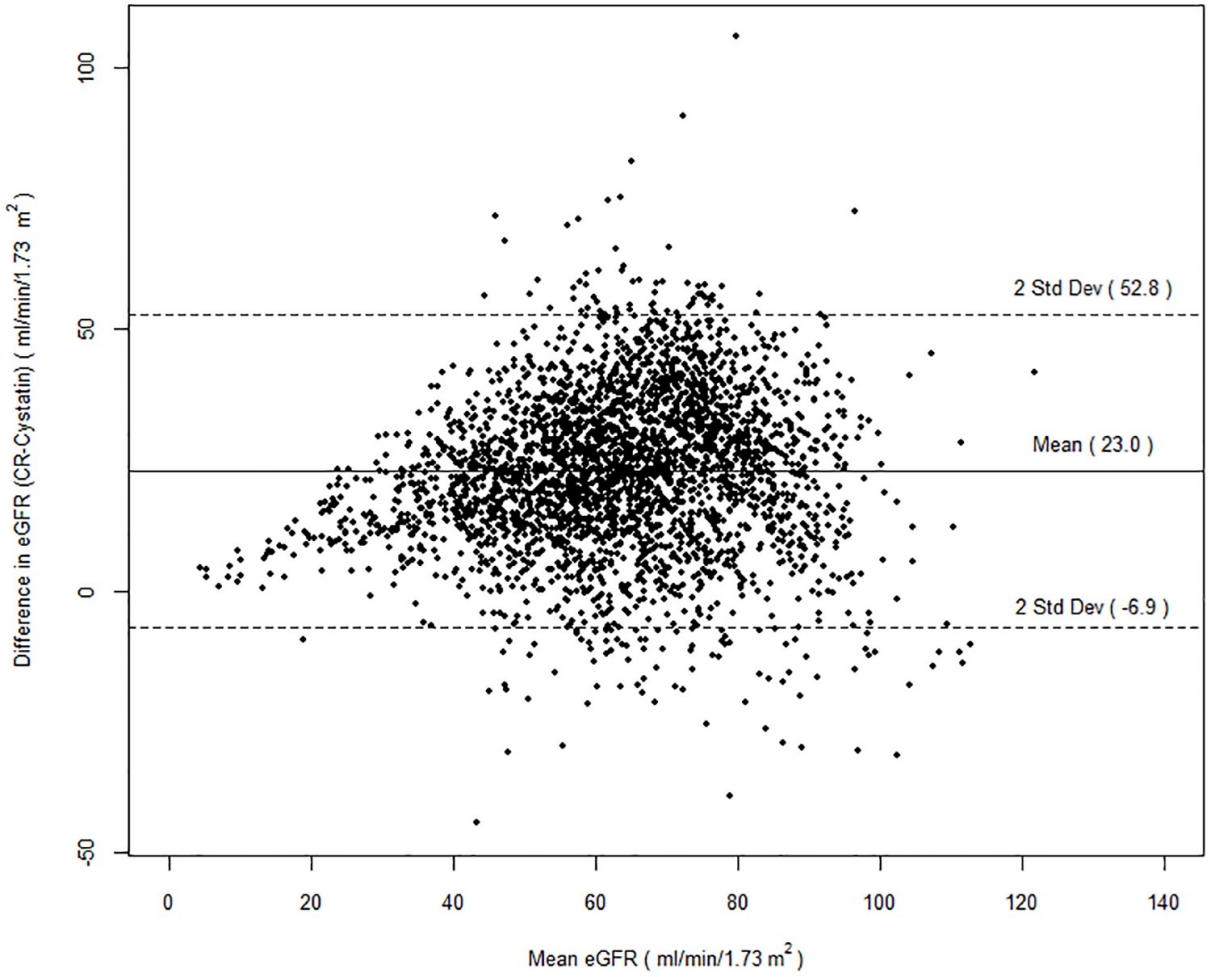
Bland-Altman plot of the difference in creatinine-based estimated glomerular filtration rate (eGFR_cr_) and cystatin C-based estimated glomerular filtration rate (eGFR_cys_) versus the mean of the two estimates.

Mean SCr was 0.96 ± 0.4mg/dL and mean cystatin C was 1.4 ± 0.6mg/L, corresponding to mean eGFR_cr_ 75 ± 19 ml/min/1.73m^2^ and mean eGFR_cys_ 52 ± 17 ml/min/1.73m^2^. The creatinine- and cystatin-based GFR estimates (eGFRcr and eGFRcys, respectively) were correlated (r = 0.62, p<0.001) (S3 and S4 Figs). On average, eGFRcys was 23±15ml/min/1.73m2 lower than eGFRcr. The Bland-Altman plot shows that the participants primarily displayed difference between eGFRcr and eGFRcys of -7 to +53 (mean ± 2 standard deviations) with decreasing agreement noted at higher mean eGFR (Fig 1). Accordingly, there was a markedly higher prevalence of CKD (eGFR<60ml/min/1.73m^2^), using eGFR_cys_ compared to eGFR_cr_ (71% vs 22%, p<0.001) (Tables 2 and 3).

**Table 2:** Comparison of CKD diagnosis by equation.

eGFR (SCr)	eGFR (Cys)		
	≥ 60	< 60	Total
≥ 60	**838**	**1495**	2333
	**(28.1%)**	**(50%)**	(78.1%)
< 60	**43**	**612**	655
	**(1.4%)**	**(20.5%)**	(21.9%)
Total	881	2107	2988
	(29.5%)	(70.5%)	

CKD = chronic kidney disease, eGFR = estimated glomerular filtration rate

**Table 3:** Distribution of CKD stage by GFR-estimating equation.

eGFR_cr_	eGFR_cys_					Total	
	<15	15–29	30–59	60–89	≥90	N	%
<15	**11** (92%)	**1** (8%)	**0**	**0**	**0**	12	0.4
15–29	**19** (56%)	**14** (41%)	**0**	**1** (3%)	**0**	34	1.1
30–59	**10** (2%)	**172** (28%)	**385** (63%)	**40** (7%)	**1** (<1%)	608	20.4
60–89	**2** (<1%)	**48** (3%)	**1181** (72%)	**380** (23%)	**19** (1%)	1630	54.5
≥ 90	**0**	**8** (1%)	**256** (36%)	**402** (57%)	**38** (5%)	704	23.6
Total	42	243	1822	823	58	2988	
%	1.4	8.1	61	27.5	1.9		

CKD = chronic kidney disease, eGFR = estimated glomerular filtration rate

The proportion of discordance between eGFR_cr_ and eGFR_cys_-based CKD diagnosis was 51%. The highest discordance was observed among participants with eGFR_cr_≥60ml/min/1.73m^2^. Over half of the participants who were reclassified from eGFR_cr_≥60ml/min/1.73 m^2^ to eGFR_cys_<60ml/min/1.73 m^2^ had a difference in GFR estimates >30ml/min/1.73m^2^, while only 2.8% of participants displayed a difference ≤10ml/min/1.73m^2^ (Table 4). Only 43 participants had eGFR_cr_<60ml/min/1.73 m^2^ but eGFR_cys_>60ml/min/1.73 m^2^; manual review demonstrated that their GFR estimates were clustered around 60, with the majority (81%) displaying eGFR_cr_ 50–59.9 but eGFR_cys_ 60–69.9 (median eGFR difference 9.9ml/min/1.73m^2^).

**Table 4:** Distribution of eGFR discrepancy among those reclassified to CKD (i.e eGFRcr≥60 & eGFRcys<60).

eGFR_cr_—eGFR_cys_	Frequency	Percent
0–10	42	3%
10–20	249	17%
20–30	428	29%
30–40	434	29%
40–50	247	17%
50–60	80	5%
60–70	8	0.50%
>70	7	0.50%

CKD = chronic kidney disease, eGFR = estimated glomerular filtration rate

49% of participants demonstrated CKD diagnosis concordance: 21% of the cohort had evidence of CKD by both estimates, while 45 patients (1.5% of the cohort) had evidence of severe CKD (eGFR<30ml/min/1.73m^2^) using both estimates. A minority of participants (28%) maintained the same CKD staging-based eGFR categories (ie. <15, 15–29, 30–59, 60–89, ≥90) using both estimates (Tables 2 and 3).

### Reclassification among eGFR_cr_ ≥60ml/min/1.73m^2^

Among those with eGFR_cr_≥60ml/min/1.73m^2^, the proportion of reclassification (eGFR_cys_<60ml/min/1.73 m^2^) was 64%. Reclassification was not limited to participants with borderline eGFR_cr_—although those with eGFR_cr_ 60-89ml/min/1.73m^2^ were most likely to be reclassified (76%), many participants with eGFR_cr_≥90ml/min/1.73m^2^ were reclassified as well (38%). However, almost all (96%) of those reclassified were estimated to have eGFR_cys_ 30-59ml/min/1.73m^2^. Most (81%) reclassified participants had ≥20mL/min/1.73m^2^ discrepancy between GFR estimates.

We examined the association of demographics and comorbidities with the proportion of reclassification (Table 5). In an adjusted model, the odds of reclassification were greater in those with age >65 years (vs. age ≤65, OR 5.67, 95% CI 4.61–6.99), obesity (OR 2.06 vs BMI≤30, 95% CI 1.64–2.59), current smokers (OR 1.66 vs non-smokers, 95% CI 1.26–2.18), non-diabetics (OR 1.64, 95% CI 1.30–2.08), females (OR 1.56, 95% CI 1.27–1.92), and white race (vs. African American, OR 2.04, 95% CI 1.49–2.78) (vs. Hispanic, OR 1.56, 95% CI 1.18–2.08).

**Table 5:** Factors associated with reclassification to eGFR_cys_ <60 among participants with eGFR_cr_≥60.

Parameter	Adjusted*		
	OR	95% CI	
Age>65	**5.67**	**4.61**	**6.99**
Female (vs Male)	**1.56**	**1.27**	**1.92**
African American (vs. White)	**0.49**	**0.36**	**0.67**
Hispanic (vs. White)	**0.64**	**0.48**	**0.85**
Diabetics	**0.61**	**0.48**	**0.77**
Hypertension	1.24	1.00	1.54
Obese	**2.06**	**1.63**	**2.59**
Past Smoker (vs. non-smoker)	1.08	0.87	1.35
Current Smoker (vs. non-smoker)	**1.66**	**1.26**	**2.18**
Hypercholesterolemia	0.93	0.76	1.14
Any Cardiac Disease	**1.73**	**1.35**	**2.23**

OR = odds ratio, CI = confidence interval;

*Adjusted for age, sex, race-ethnicity, education, medical insurance, diabetes, hypertension, BMI, smoking status, hypercholesterolemia and cardiac disease

### Sensitivity analyses

Applying the calibration factor to serum creatinine values as described above yielded similar reclassification frequency (S1 Table). Next, we limited the analysis to participants aged <65 years with white race. These 110 participants continued to display a high frequency of reclassification: among the 88% of this subpopulation with eGFR_cr_≥60ml/min/1.73m^2^, 49% were reclassified to eGFR_cys_<60ml/min/1.73m^2^ (S2 Table). Finally, given the large difference in CKD prevalence observed above, we calculated eGFR_cr-cys_ using the CKD-EPI 2012 formula that utilizes both biomarkers in a post-hoc analysis. Expectedly, we found that this method of GFR estimation yielded a CKD prevalence in between those provided using eGFR_cr_ and eGFR_cys_ (54.8%) (S3 Table).

## Discussion

We compared creatinine- and cystatin-based GFR estimation in a large, elderly, racially/ethnically diverse cohort and found a large difference in the prevalence of CKD (defined as eGFR<60ml/min/1.73m^2^). While the direction of the findings was consistent with our hypothesis, the magnitude of the differences was striking. Over half of the participants did not retain the same CKD status using both GFR estimates, and the differences in CKD classification using eGFR_cys_ and eGFR_cr_ were not simply clustered around CKD class thresholds. In fact, even among participants with eGFR_cr_≥90ml/min/1.73m^2^ (classically considered a “normal” GFR), 37.5% were reclassified as having CKD using eGFR_cys_. This degree of discordance is greater than has been observed in other cohorts and raises potential concerns about the commonly used creatinine- and cystatin-based GFR estimates. The widely-used CKD-EPI eGFR equations were developed in a population with a median age of 47 years and only <4% of participants were aged ≥71 years.[28] In contrast, the mean participant age at the time of enrollment in NOMAS was 69 years. Further, over half of our participants self-identified as Hispanic. The cystatin C-based CKD-EPI eGFR equation may have an advantage in our cohort given the absence of inclusion of race and a smaller impact of age on the estimate.[10, 14]

Previous studies have compared creatinine- and cystatin-based eGFR in different elderly groups but have not demonstrated the degree of discordance that we observed.[29–32] The Sacramento Area Latino Study on Aging (SALSA), a cohort of Mexican-American participants with mean age 71 years, demonstrated that 10% of participants had discordant eGFR_cr_ vs eGFR_cys_ CKD classification.[29] However, the SALSA cohort had a much lower prevalence of CKD compared to the NOMAS cohort, with only 21% of participants having CKD based on any marker (whereas in our group 22% and 71% had CKD based on creatinine and cystatin C, respectively).[29] A smaller study comparing GFR estimates in 95 elderly Brazilian participants Brazil (mean age 85.3 years) suggested that although eGFR_cr_ overestimated GFR, eGFR_cys_ was more biased than eGFR_cr_, and the combined use of both markers performed better.[33] Similarly, estimating GFR with a combined creatinine and cystatin equation outperformed eGFR_cys_ and eGFR_cr_ in an elderly Icelandic cohort (n = 805, mean age 80.3 years).[32] A portion of another analysis with participants of primarily European ancestry (n = 394, mean age 80 years) found that while both equations overestimated GFR in those without decreased measured GFR, eGFR_cys_ tended to underestimate GFR in patients with measured GFR<60ml/min/1.73m^2^, while eGFR_cr_ overestimated GFR in the same group.[34] An additional study examined reclassification among participants in the Osteoporotic Fractures in Men Study (mean age 76.4 years) and found a large discrepancy between prevalence of eGFR<60ml/min/1.73m^2^ based on eGFR_cys_ and eGFR_cr_ (36% vs 23%), with eGFR_cys_ providing superior prediction of mortality.[35]

The magnitude of the difference between the equations demonstrated in our study is large and, given the implications for incorrect medication dosing, particularly concerning. Our prevalence of eGFR_cys_-based CKD is consistent with prior data that used the creatinine-based Modification of Diet in Renal Disease (MDRD) Study formula and noted prevalent CKD Stages 1–4 in almost half of NHANES 1999–2004 participants age ≥70 years, with 37.8% of participants in this age group having CKD stages 3 or 4.[36, 37] Further, that analysis also showed a significant increase in CKD prevalence over time, supporting the plausibility of our findings.[36] The large discrepancies we observed in the absence of measured GFR data (using an exogenous marker) as a reference suggest that either one or both estimates lose precision in our elderly cohort. Several potential contributing factors should be considered as possible explanations.

First is a possible systematic *over*estimation of GFR using eGFR_cr_ in our cohort. In our cohort, age was associated with reclassification of creatinine-based CKD status using cystatin C, whereas male sex was associated with decreased likelihood of reclassification. Prior investigators have suggested using age, cardiovascular disease, diabetes, hypertension, obesity, and smoking to identify patients who may have occult CKD not identified using eGFR_cr_ alone and therefore warrant eGFR_cys_ assessment.[38] With the exception of diabetes, our data is consistent with these prior findings. An increased prevalence of sarcopenia with age may be contributing to lower-than-expected serum creatinine independent of renal function and lead to the large discrepancy between eGFR_cr_ and eGFR_cys_ that we observed.

The other possibility is that eGFR_cys_ systematically *under*estimates GFR in the NOMAS cohort instead of (or in addition to) the hypothesized mechanism above. Age, weight, and smoking are known to be associated with increased cystatin C levels even after controlling for creatinine clearance.[39] Consistent with this, these patient characteristics were all associated with increased reclassification in our cohort supporting the notion that a high prevalence of obesity and smoking would limit the ability of eGFR_cys_ to accurately identify CKD in older cohorts.

Finally, currently available cystatin C assays vary significantly, underscoring the need for the ongoing development of reference material for cystatin C assays intended to improve this problem.[40–46] In contrast to the CKD-EPI group, which used a Siemens Dade Behring Nephelometer traceable to IFCC/IRMM reference materials, we used a Roche assay also standardized against ERM-DA471/IFCC reference material. Despite this difference, the cystatin C values we observed are quite plausible. The mean serum cystatin C was 1.4mg/L in the CKD-EPI study development/internal validation cohort.[10] Further, a previous investigation on cystatin C concentration in healthy elderly subjects (age ≥65 years) in Britain found a mean cystatin C of 1.48mg/L in females and 1.53mg/L in males, also similar to our observed mean of 1.4mg/L.[47] Other prior studies focusing elderly participants have demonstrated variable mean cystatin C values, ranging from 1.14–1.44 mg/L.[21, 32–34] While it is certainly possible that our cystatin C assay may be less reliable than expected, considering that our testing utilized a commercially-available cystatin C assay, these discrepancies underscore the need for further studies including measured GFR data to determine their etiology in addition to the need for better cystatin assay standardization. This uncertainty should similarly be considered when interpreting GFR estimates that utilize both creatinine and cystatin (eGFR_cr-cys_), and additional investigation is needed to determine whether the use of both biomarkers together improves the accuracy of GFR estimates in this population.

Grubb, et al have proposed an alternative, assay-independent, cystatin-based eGFR equation (“CAPA”) developed using Swedish, Japanese, and Dutch cohorts.[24] However, given the limited demographic subgroups in which this equation was developed, its applicability to a broad section of the United States population remains uncertain. Similarly, the BIS (Berlin Initiative Study) equations designed for use in the elderly included only subjects living in Berlin, Germany.[21] Domestic initiatives are warranted to better address the need for improved GFR assessment in the United States, particularly in non-White populations.

Our findings have significant implications for clinical practice because the discrepant CKD categorizations would warrant different dose adjustments and carry significantly different prognostic implications. Currently, clinicians routinely receive eGFR data alongside serum creatinine results. Our observations suggest that these data could be misleading in populations similar to ours, potentially leading to inappropriate decisions regarding drug dosing, drug safety, risk stratification, and eligibility for advanced therapeutics. Accurate GFR ascertainment is critical for accurate dosing of medications cleared by the kidney, especially among elderly patients already at higher risk of polypharmacy-associated complications. This underscores the need for further study of the performance of different methods of GFR estimation in older, diverse populations.

In addition to changing the need for evaluation of CKD-associated complications including anemia and bone disease, accurately identifying decreased GFR can help clinicians better assess the contribution of CKD to patient outcomes such as cardiovascular events and mortality. Discrepant estimates using creatinine and cystatin may even be a marker for sarcopenia in the elderly and thus further inform the clinical care of these patients.[48] Finally, the timely CKD diagnosis and early referral to a nephrologist have been associated with improved outcomes.[49]

Our study confirms previous findings of significantly discrepant CKD diagnosis in elderly patients, but in a larger multiethnic cohort. The strengths of our study include a large sample size and diverse population. Limitations include the lack of measured GFR testing, as detailed above. These data would allow us to determine whether the differences between eGFR_cr_ and eGFR_cys_ result from lack of precision of one or both GFR estimates. Additionally, we lack information regarding albuminuria, an important component of CKD diagnosis, classification, and risk prediction, and our cross-sectional design relies on a single simultaneous creatinine and cystatin measurements for each participant. It should also be noted that the use of concordance of eGFR<60ml/min/1.73m^2^ (i.e. the established eGFR-based threshold for CKD diagnosis) as a primary endpoint is somewhat arbitrary, although the large differences in GFR estimates we observed emphasize the clinical importance of these findings. Further studies investigating differences in morbidity and mortality, as well as the development of clinically relevant kidney disease, among our participants whose CKD status was reclassified are also warranted, as the value of precise GFR estimates may not necessarily translate to improved prediction of adverse outcomes such as progression to end-stage renal disease or incidence of cardiovascular events.

## Conclusions

In a large, racially/ethnically diverse, elderly population, we observed a dramatic increase in the prevalence of CKD and a significant amount of reclassification of CKD diagnosis when using cystatin C- rather than creatinine-based estimates of GFR. Given the aging of the population and the known associations between CKD and adverse cardiovascular events, our findings suggest that urgent further study is needed to clarify the accuracy of different methods of GFR estimation in the elderly. Improving GFR estimation in elderly patients can help identify unrecognized CKD, prevent CKD misdiagnosis, and promote management of CKD-associated complications at an earlier stage.

## Supporting information

S1 Fig Difference between measured creatinine and standardized creatinine after creatinine calibration to IDMS standard. (TIF)

S2 Fig Measured creatinine vs standardized creatinine after creatinine calibration to IDMS standard. (TIF)

S3 Fig Serum creatinine (mg/dL) versus serum cystatin C (mg/L). (TIF)

S4 Fig eGFR_cr_ (ml/min/1.73m^2^) vs eGFR_cys_ (ml/min/1.73m^2^). (TIF)

S1 Table Sensitivity analysis- distribution of CKD diagnosis by GFR-estimating equation using calibrated creatinine. (DOCX)

S2 Table Sensitivity analysis- distribution of CKD diagnosis by GFR-estimating equation in white participants with age <65 years. (DOCX)

S3 Table Prevalence of eGFR<60ml/min/1.73m^2^ based on creatinine, cystatin, and combined creatinine-cystatin GFR estimation. (DOCX)
