# Supplementary figures and images for: Creatinine- versus cystatin C-based renal function assessment in the Northern Manhattan Study
Source: PLoS One. 2018 Nov 14;13(11):e0206839. doi: 10.1371/journal.pone.0206839 (PMC6235352; doi:10.1371/journal.pone.0206839)

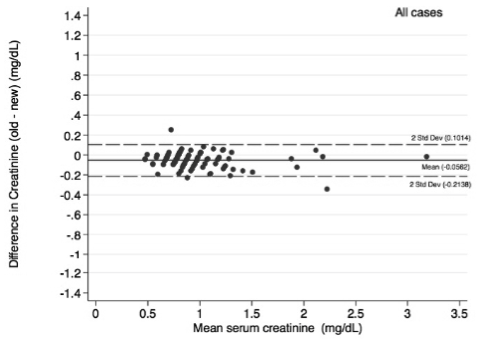

Supplement: S1 Fig — (TIF) [file pone.0206839.s001.tif]

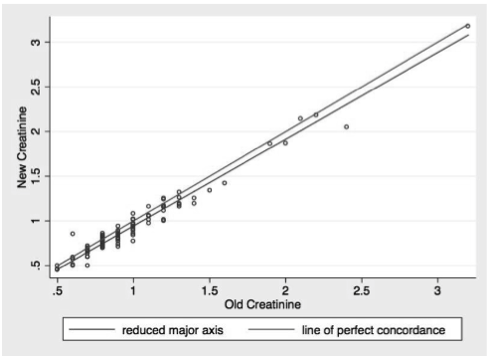

Supplement: S2 Fig — (TIF) [file pone.0206839.s002.tif]

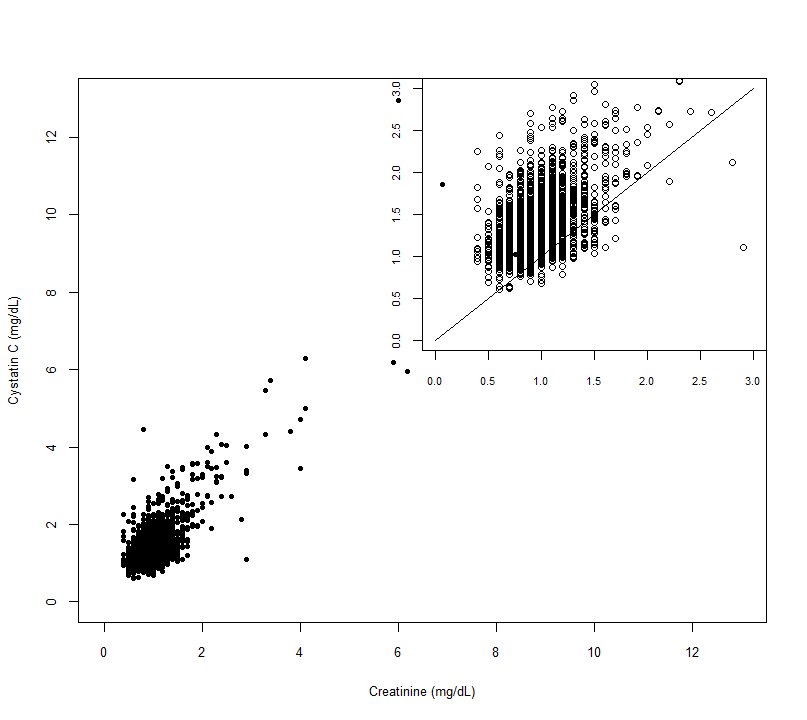

Supplement: S3 Fig — (TIF) [file pone.0206839.s003.tif]

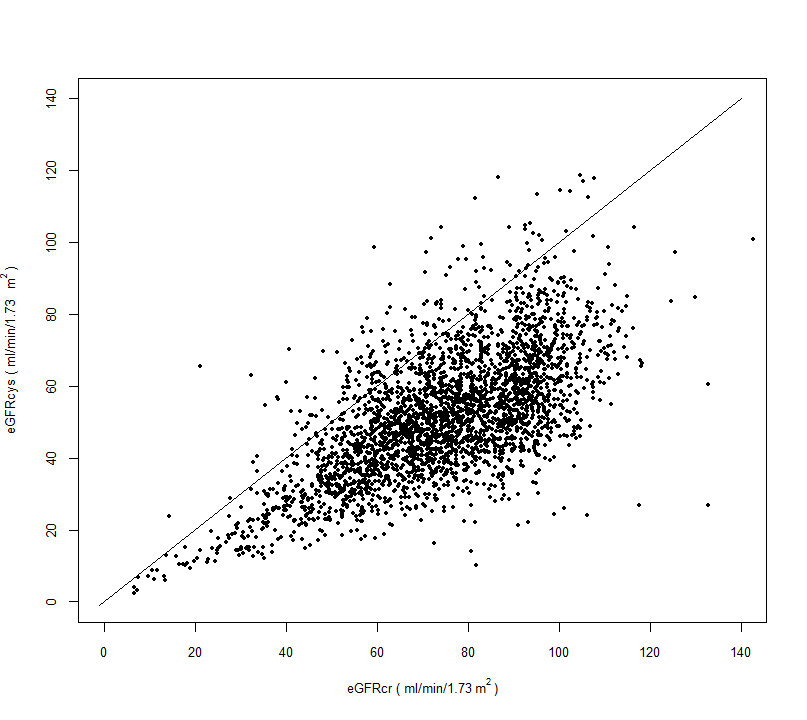

Supplement: S4 Fig — (TIF) [file pone.0206839.s004.tif]
