# Supplementary material for: Creatinine- versus cystatin C-based renal function assessment in the Northern Manhattan Study
Source: PLoS One. 2018 Nov 14;13(11):e0206839. doi: 10.1371/journal.pone.0206839 (PMC6235352; doi:10.1371/journal.pone.0206839)
Supplement: S1 Table — (DOCX) [file pone.0206839.s005.docx]

**Supplemental Table 1: Sensitivity Analysis- Distribution of CKD Diagnosis by GFR-estimating Equation using Calibrated Creatinine**

| eGFR_cr_ | eGFR_cys_ | | |
| --- | --- | --- | --- |
|  | ≥ 60 | < 60 | Total |
| ≥ 60 | 862 | 1643 | 2505 |
|  | 29% | 55% | 84% |
| < 60 | 19 | 464 | 483 |
|  | <1% | 16% | 16% |
| Total | 881 | 2107 | 2988 |
|  | 29% | 71% | 100% |
