# Supplementary material for: Creatinine- versus cystatin C-based renal function assessment in the Northern Manhattan Study
Source: PLoS One. 2018 Nov 14;13(11):e0206839. doi: 10.1371/journal.pone.0206839 (PMC6235352; doi:10.1371/journal.pone.0206839)
Supplement: S2 Table — (DOCX) [file pone.0206839.s006.docx]

**Supplemental Table 2: Sensitivity Analysis- Distribution of CKD Diagnosis by GFR-estimating Equation in White Participants with Age <65 years**

| eGFR_cr_ | eGFR_cys_ | | |
| --- | --- | --- | --- |
|  | ≥ 60 | <60 | Total |
| ≥ 60 | 49 | 48 | 97 |
|  | 45% | 44% | 88% |
| < 60 | 2 | 11 | 13 |
|  | 2% | 10% | 12% |
| Total | 51 | 59 | 110 |
|  | 46% | 54% | 100% |
