# Supplementary material for: Creatinine- versus cystatin C-based renal function assessment in the Northern Manhattan Study
Source: PLoS One. 2018 Nov 14;13(11):e0206839. doi: 10.1371/journal.pone.0206839 (PMC6235352; doi:10.1371/journal.pone.0206839)
Supplement: S3 Table — (DOCX) [file pone.0206839.s007.docx]

Supplemental Table 3: Prevalence of eGFR<60ml/min/1.73m^2^ based on creatinine, cystatin, and combined creatinine-cystatin GFR estimation

|  | **eGFR (ml/min/1.73m^2^)** | |
| --- | --- | --- |
|  | **≥60** | **<60** |
| **eGFR_cr_** | 78.1% | 21.9% |
| **eGFR_cys_** | 29.5% | 70.5% |
| **eGFR_cr-cys_** | 54.8% | 45.3% |
